# Is a fusobacterium nucleatum infection in the colon a risk factor for colorectal cancer?: a systematic review and meta-analysis protocol

**DOI:** 10.1186/s13643-019-1031-7

**Published:** 2019-05-10

**Authors:** Amal Idrissi Janati, Igor Karp, Hisham Sabri, Elham Emami

**Affiliations:** 1 Faculté de Médecine Dentaire, Université de Montréal, C.P. 6128, Succursale Centre-Ville, Montréal, Québec H3C 3J7 Canada; 2 Department of Epidemiology and Biostatistics, Schulich School of Medicine and Dentistry, University of Western Ontario, 1151, Richmond St., Kresge Building, Room K214, London, Ontario N6A 5C1 Canada; 3 Department of Social and Preventive Medicine, School of Public Health, Université de Montréal, Montreal, Canada; 4 Department of Psychology, Concordia University, Montreal, Canada; 5 Faculty of Dentistry, McGill University, Montreal, Canada; 6 Department of Restorative Dentistry, Faculty of Dentistry, McGill University, 2001 McGill College Avenue, Suite 500, Montreal, QC H3A 1G1 Canada

**Keywords:** Colorectal cancer, Fusobacterium nucleatum, Systematic review protocol, Meta-analysis

## Abstract

**Background:**

Despite a considerable amount of epidemiological research for identification of risk factors involved in the development of colorectal cancer, the current understanding of the etiology of this disease remains rather poor.

Accumulating evidence suggests a potentially important role of infection with Fusobacterium nucleatum in the colon in colorectal carcinogenesis. The objective of this systematic review is to synthesize the epidemiological evidence on the association between infection with Fusobacterium nucleatum in the colon and colorectal cancer.

**Methods:**

This systematic review will include observational studies (cohort, case**-**control, cross-sectional) in humans in which the role of Fusobacterium nucleatum in the etiology of colorectal cancer was investigated. MEDLINE, EMBASE, Web of Science, and Cochrane Database of Systematic Reviews will be searched using a comprehensive search strategy and manual screening of references. Two reviewers will independently identify eligible studies and extract the data from the included studies. The quality of studies will be assessed by using the Newcastle-Ottawa scale. Random-effects models will be used to estimate pooled measures of association (where feasible). Meta-regression and subgroup analyses will be conducted to explore the potential sources of heterogeneity. The Meta-Analysis of Observational Studies in Epidemiology (MOOSE) guidelines and the Preferred Reporting Items for Systematic Reviews and Meta-Analyses (PRISMA) statement will be followed for reporting.

**Discussion:**

Deepening knowledge regarding the etiology of colorectal cancer and the potential implications of Fusobacterium nucleatum in this disease is instrumental for prevention, diagnosis, and treatment of this often-fatal disease. This review will produce summarized current evidence on this topic.

**Systematic review registration:**

This systematic review protocol has been registered with the International Prospective Register of Systematic Reviews (PROSPERO) on 10 July 2018 (registration number CRD42018095866).

**Electronic supplementary material:**

The online version of this article (10.1186/s13643-019-1031-7) contains supplementary material, which is available to authorized users.

## Background

### Rationale

In 2012, over a million new cases of colorectal cancer (CRC) and more than half a million deaths due to CRC were estimated to occur globally [1]. Of cancers that affect both men and women, CRC is the third most commonly diagnosed malignancy and the fourth most fatal in the world [2]. The numbers of new cases of CRC and CRC deaths are expected to increase to 60% by 2030 [2].

The etiology of CRC is widely recognized as being multifactorial [3, 4], and previous research has suggested that modification of environmental and lifestyle factors can lead to important changes in cancer risk [5, 6]. Still, according to comprehensive reviews of the available evidence conducted by expert panels from the American Institute for Cancer Research and the World Cancer Research Fund, the overall evidence for the causal nature of the association with CRC is considered convincing for only some of the previously suggested factors, namely, excess body fat, consuming processed meats and red meat, physical inactivity, cigarette smoking, and alcohol consumption [7]. For the majority of the putative risk factors, the level of evidence is considered either fair or inadequate [7–10]. Thus, identification of modifiable risk factors that could serve as targets for preventive interventions is a current public-health priority.

In the past few years, advances in high-throughput sequencing technologies have led to important discoveries on the role of gut microbial dysbiosis and specifically, of Fusobacterium nucleatum (F*. nucleatum*) in colorectal carcinogenesis [11–21]. F*. nucleatum* is a Gram-negative, non-spore-forming anaerobic bacterium commonly found in saliva and oral biofilm [18, 22, 23] . It is one of the dominant species of more than 500 organisms of the oral cavity and has five subspecies with different specific genome sequences [24–31]. This invasive proinflammatory agent is involved in the pathogenesis of periodontal diseases [22] as well as of other oral [32] and extra-oral infections [33, 34]. F*. nucleatum* can independently invade host cells via surface adhesins and invasion molecules such as FadA [21, 35]. Importantly, once disseminated outside the oral cavity, FadA activates proinflammatory and oncogenic signals and stimulates the growth of epithelial cells. Human studies have demonstrated that the FadA gene level in CRC tissue is higher than in normal tissue and is correlated with expression of inflammatory genes [21]. Furthermore, a recent study found a strong correlation between F*. nucleatum* and proinflammatory markers such as COX-2, IL-8, IL-6, IL1ß, and TNF-α in CRC [15]. This evidence suggests that colonization resistance of the healthy gut can be disrupted by bacterial species that trigger a systematic inflammatory response, such as seen in periodontal disease. In a study by Dejea et al. [36], the rate of CRC occurrence was more than five times as high in individuals with gut bacterial biofilms as in those without them [36]. Interestingly, the gut bacterial biofilm composition and invasiveness were similar to those found in oral biofilm in periodontal disease, with *Fusobacteria* being a dominant species [36].

F*. nucleatum* is now considered to be a pathogenic bacterium of the gut that can invade the colorectal submucosa and epithelium. Various studies have shown an overabundance of F*. nucleatum* in tumors and fecal samples [37] of CRC patients [15, 17, 19–21, 38] . Additionally, some studies have demonstrated that levels of F. *nucleatum* increased in parallel with the transition from healthy colorectal tissue to adenomas and finally to CRC [39–41]. F. *nucleatum* levels in cancerous colorectal tissue have also been shown to serve as a prognostic indicator in CRC [11, 39, 42]. In vitro and in vivo studies showed that F*. nucleatum* interrupts oncogene signaling and cell–cell adhesion and inhibits the anti-tumor activities of natural killer and cytotoxic T cells as well as anti-tumor immunity [38, 43]. Increased levels of F*. nucleatum* have been shown to be associated with microsatellite instability and molecular subsets of CRCs such as the CpG island methylator phenotype [11, 44]. Decreased expression of MLH1, a primary cause of microsatellite instability, was found in samples abundant in F. *nucleatum* [13, 42]. Other markers of poor prognosis such as KRAS and BRAF are also overexpressed in samples rich in F. *nucleatum* [13, 45, 46]. Moreover, CRC patients have been found to have an increased level of serum anti-F*. nucleatum* antibodies [47].

The literature on the association between F*. nucleatum* and CRC is growing but has not yet been systematically reviewed to date. We aim to conduct a systematic review of observational studies on the association between F. *nucleatum* and CRC.

### Objectives

The aim of this review is to systematically identify, review, and assess the quality of available literature on the association between F*. nucleatum* and CRC. The findings of this systematic review will help answer the following question: does F*. nucleatum* play a role in the etiology of CRC? If feasible, a meta-analysis will be conducted to estimate pooled measures of association between F. *nucleatum* and CRC.

## Methods

The protocol has been developed in accordance with the preferred reporting items for systematic review and meta-analysis protocols 2015 (PRISMA-P) [48, 49], which is available in Additional file 1. However, as at issue in this review is the topic of disease etiology (rather than intervention effects), the PICO format will be replaced by PECO (population, exposure, control, outcome), as detailed in the MOOSE (the Meta-analysis of Observational Studies in Epidemiology) guidelines [50].

### Eligibility criteria

Studies will be selected according to the following criteria:

#### Study design

Original observational research studies that address the association between F*. nucleatum* and CRC will be included in this review. This includes cohort studies, case-control studies, and cross-sectional studies. Case reports, position papers, and reviews will be excluded from the current review.

#### Participants/population

The population of interest will be unrestricted in terms of age, sex, ethnicity, socioeconomic status, occupation, or history of other diseases.

#### Exposure

The review will consider the exposure of interest, the F*. nucleatum* infection in the colon. Ascertainment of this infection should be based on tests such as quantitative polymerase chain reaction (qPCR), 16S rRNA gene sequencing, or fluorescence in situ hybridization (FISH).

#### Comparator

The comparator category will be the absence of the F*. nucleatum* infection in the colon.

#### Outcomes

The outcome of interest will be the presence of CRC, based on clinically confirmed diagnosis (i.e., cancer registry, hospital, or doctors’ records).

#### Setting and language

There will be no restriction by study setting. English- and French-language publications will be considered for full-text analysis in this systematic review, and eligible articles in other languages will be translated using Google Translate.

### Information sources

An electronic literature search will be conducted in the following databases: MEDLINE (OVID interface, 1946 onwards), EMBASE (OVID interface, 1974 onwards), Web of Science, and Cochrane Database of Systematic Reviews (CDSR). Since the vast majority of research conducted on this topic has been carried out in the past 10 years, the start date for the literature search will not be limited in order to maximize the number of publications considered. The electronic literature search will be complemented by hand searching the list of references in the identified publications or relevant reviews. NICE Evidence and TRIP database will be searched for gray literature using subject keywords. Ongoing studies (which have not resulted in publications on the topic at issue) will not be considered in the review.

### Search strategy

Using medical subject headings (MeSH), EMTREEs and text words related to the field of the study, the research team has developed a draft version of literature search strategies with the help of an expert librarian at Université de Montréal (Additional file 2). This draft of the MEDLINE search strategy will be finalized and adapted to the other databases using the proper syntax, subject headings, and controlled vocabulary considering maximized sensitivity of the search. In order to maximize the yield of the search strategy, no language restrictions in the search strategy will be used. Hand searching the list of references in the identified publications, NICE Evidence and TRIP database will be done to identify the relevant articles in the gray literature. The PROSPERO will be searched for recently completed systematic reviews on the topic. The references of relevant studies will be verified for relevant publications.

### Study records

#### Data management, selection, and data collection process

Data will be collected using EndNote software and a pre-designed data collection form. The reliability of data selection process will be pilot tested in 10% of randomly included articles, and Cohen’s kappa will be calculated to assess the inter-reviewer (AIJ, EE) agreement on study eligibility. Two independent reviewers (AIJ, EE) will screen all retrieved titles and abstracts using the eligibility criteria. In the case of incomplete information provided by the title and abstract, the full text will be used to determine the study’s eligibility to be included for the study. In the case of multiple reports of the same study, the most recent article will be included in the review.

Disagreements between the two reviewers will be resolved by discussion and resolved through consensus-seeking. If an agreement cannot be achieved, the opinion of a third reviewer (IK) will be sought.

#### Data items

The data will be extracted independently by two reviewers (AIJ, EE) from the full text of the included studies. The extracted information will include authors, country, year of publication, aim of the study, study design, sample size, study participants’ characteristics (age, sex, the stage of CRC for cases), study population, exposure description, the technique used to quantify the exposure, type and number of controls, and the number of *F. nucleaum*-positive, as well as the main results. In the case of insufficient data, we will contact authors via email for additional information. If the missing data cannot be rectified by author contact, we will use narrative approaches to describe the major findings.

### Outcomes and prioritization

As stated in the outcomes section, colorectal cancer will be the study outcome.

### Risk of bias and level of evidence

Two reviewers will independently assess the risk of bias of the eligible studies using the Newcastle-Ottawa scale (NOS) [51]. This 8-item scale allows to assess the quality of the articles based on the selection of the study groups; the comparability of the groups; and the ascertainment of either the exposure or outcome of interest for case-control or cohort studies, respectively. Disagreement will be resolved by consultation with a third reviewer.

We will also evaluate the level of evidence of all studies according to the Oxford Level of Evidence [52].

## Data synthesis

We will use two approaches for data synthesis. The descriptive synthesis will be conducted according to the Centre for Reviews and Dissemination [53] and will include text and tables to summarize the characteristics of the findings and explain the findings. Where feasible (availability of two or more studies with similar study design, estimates of ‘relative’ measures of association (risk ratio, incidence-odds ratio, rate ratio, hazard ratio, prevalence ratio, prevalence-odds ratio) and their corresponding measures of imprecision (standard error, confidence interval)), the data will be pooled using a random-effects model.

Heterogeneity across studies will be tested using Cochran’s Q and the *I*^2^ statistic. To examine the potential sources of heterogeneity (due to personal characteristics, study design, etc.) across the studies, subgroup analyses and meta-regression will be conducted. We will use the Comprehensive Meta-Analysis Version 2 to conduct the meta-analysis [4].

### Meta-biases

The potential publication bias will be assessed by funnel plots [54]. Tests for funnel plot asymmetry will be conducted if the number of studies included in the meta-analysis is more than 10 [55].

### Confidence in cumulative evidence

The Oxford Level of Evidence [56, 57] and The Grading of Recommendations Assessment, Development, and Evaluation (GRADE) approach will be used to evaluate the level of evidence of all studies.

### Differences between the protocol and the review

Any deviations from the protocol due to unanticipated issues will be reported in the final review [58].

## Discussion

This systematic review will assess the role of F*. nucleatum* in the etiology of CRC. Specifically, we will identify, assess, and synthesize the available evidence from published observational studies on the role played by F*. nucleatum infestation* in the colon in the development of CRC.

Caution will have to be taken when interpreting the results of this systematic review. We are aware that observational studies are subject to a high risk of bias due to potential outcome confounding. In addition, if the heterogeneity of the studies is high, this may preclude obtaining a meaningful pooled estimate of the association of interest (or introduce challenges in interpreting a pooled estimate of the association).

Despite the above limitations, we expect that the systematic review proposed here will be of high scientific and pragmatic value. The study aims to facilitate achieving a comprehensive and up-to-date understanding of the association between F*. nucleatum* and CRC. To our knowledge, our systematic review will be the first to synthesize the available evidence on this association. Findings from this study could help pave the way for the development of new methods of prevention, diagnosis, and treatment of CRC.

## Additional files

Additional file 1: PRISMA**-**P 2015 Checklist. (DOCX 35 kb)

Additional file 2: MEDLINE search strategy. (DOCX 13 kb)

## Abbreviations

CDSR: Cochrane Database of Systematic Reviews
CRC: Colorectal cancer
F*. nucleatum*: Fusobacterium nucleatum
FISH: Fluorescence in situ hybridization
GRADE: Grading of Recommendations Assessment, Development, and Evaluation
MOOSE: Meta-Analysis of Observational Studies in Epidemiology
NOS: Newcastle-Ottawa scale
PRISMA: Preferred Reporting Items for Systematic Reviews and Meta-Analyses
PRISMA-P: Preferred Reporting Items for Systematic Reviews and Meta-Analyses protocols
PROSPERO: Prospective Register of Systematic Reviews
qPCR: Quantitative polymerase chain reaction
