# Supplementary material for: Is a fusobacterium nucleatum infection in the colon a risk factor for colorectal cancer?: a systematic review and meta-analysis protocol
Source: Syst Rev. 2019 May 10;8:114. doi: 10.1186/s13643-019-1031-7 (PMC6511124; doi:10.1186/s13643-019-1031-7)
Supplement: Supplementary file 2 — MEDLINE search strategy. (DOCX 13 kb) [file 13643_2019_1031_MOESM2_ESM.docx]

Additional file 2: MEDLINE search strategy

| 1 | exp Fusobacterium nucleatum/ or exp Fusobacterium Infections/ or exp fusobacteria/ or (Fusobacterium nucleatum or fusobacteria or fusobacterium infection$).tw. | 6982 |
| --- | --- | --- |
| 2 | exp Colonic Polyps/ or (intestinal polyp$ or colonic polyp$).tw. | 14860 |
| 3 | exp colonic neoplasms/ or exp rectal neoplasms/ | 102358 |
| 4 | ((colon$ or rect$ or colorect$ or bowel or large intestin$) adj5 (neoplas$ or cancer$ or carcinoma$ or tumo$ or malignan$ or adenocarcinoma$)).tw. | 210341 |
| 5 | 2 or 3 or 4 | 251367 |
| 6 | 1 and 5 | 168 |
